# Is chronological age predictable on panoramic images using the Demirjian method on third molars? Insights from a Turkish population-based study

**DOI:** 10.4317/medoral.27651

**Published:** 2025-10-17

**Authors:** Ayse Tas, Nelli Agbulut, Yasemin Oztoprak, Kader Aydin

**Affiliations:** 1 Istanbul Medipol University School of Dentistry, Department of Oral and Maxillofacial Radiology, Istanbul, Turkey; 2 Istanbul Medipol University School of Dentistry, Department of Oral and Maxillofacial Surgery, Istanbul, Turkey

## Abstract

**Background:**

Third molars are crucial in forensic age estimation during adolescence due to their late and variable development. The modified Demirjian method employs a nine-stage classification (0-H) for assessing dental maturity. However, population-specific validation is essential for forensic accuracy. This study aims to evaluate the relationship between chronological age and M3 development stages in a Turkish population using panoramic radiographs, and to investigate the influence of gender and anatomical location, including impaction classifications (Pell-Gregory and Winter).

**Material and Methods:**

A total of 1656 individuals (mean age: 13.9±3.78 years) underwent digital panoramic radiography. Developmental stages of 2770 mandibular and 2553 maxillary M3s were assessed using the modified Demirjian method. Statistical analyses included Spearman correlation, Kruskal-Wallis tests and generalized linear models.

**Results:**

Third molar development strongly correlated with chronological age. Most teeth were in stages B-E, with mandibular M3s most frequent in stages C and E, and maxillary in C and D. Age differed significantly across all stages (p&lt;0.001). Generalized linear models confirmed age as a significant predictor (R²=0.330 mandible; R²=0.337 maxilla). Gender and impaction classifications had no main effects, but significant interactions were found (Pell-Gregory×Gender: p&lt;0.001; Winter×Pell-Gregory: p=0.016).

**Conclusions:**

The modified Demirjian method reliably estimates age in Turkish adolescents, with high age-stage correlation. While gender and anatomical location alone do not alter developmental progression, their interaction with impaction patterns reveals complex growth dynamics. These findings support context-sensitive forensic protocols.

## Introduction

Accurate age estimation is essential in forensic odontology and in clinical dental practice, particularly during adolescence, when skeletal and dental indicators display significant inter-individual variability. Among all dental structures, third molars are uniquely valuable for age estimation due to their extended and late developmental timeline typically initiating mineralization around age 9 and achieving complete root apexification between 21 and 22 years (1 - 3). This protracted maturation period renders them particularly useful for estimating chronological age during late adolescence and early adulthood, especially when conventional skeletal markers have already reached maturity or are unavailable.

In clinical dentistry, third molar development plays a critical role in surgical planning. Oral and maxillofacial surgeons often rely on molar maturation stages to determine optimal timing for extraction, particularly in orthodontic, prosthodontic, and auto-transplantation cases. The eruption potential of impacted molars and the associated risk of pathologies, such as cyst formation or resorption of the adjacent teeth, further highlight the importance of accurate developmental assessment (4 , 5).

Developmental trajectories differ between mandibular and maxillary third molars due to anatomical factors such as ramus space availability, angulation, and skeletal growth patterns. These factors are further characterized by widely used classification systems such as Pell-Gregory and Winter, which aid in the assessment of impaction patterns and surgical difficulty (6). Simultaneously, radiographic staging methods, particularly the Demirjian method, are extensively employed to evaluate third molar development. Although the original Demirjian staging comprises eight stages (A-H), a modified version introduces Stage 0 to capture early follicular formation, thereby enhancing developmental assessment (7).

While the Demirjian staging has demonstrated strong correlations with age across multiple populations, its reliability may vary due to ethnic, geographic, nutritional, and socioeconomic factors. These population-specific variations may significantly influence mineralization rates, making localized validation essential to improve forensic accuracy and clinical applicability (8 - 10).

In this context, the present study aims to assess the relationship between chronological age and third molar developmental stage in a large Turkish cohort using digital panoramic radiography and the modified Demirjian method. Additionally, we investigate whether this relationship is modulated by gender and anatomical location, incorporating other classification systems to explore developmental variability in mandibular versus maxillary arches.

## Material and Methods

This retrospective study was conducted in accordance with the ethical standards of the 1964 Declaration of Helsinki and its later amendments. Approval was obtained from the Ethics Committee of the University (Decision number: 2025/682). Orthopantomographic radiographs (OPTGs) of patients referred to the university hospital between May 2021 and March 2025 were analyzed.

Inclusion criteria consisted of participants aged between 7 and 24 years, to allow for a comprehensive evaluation of the entire Demirjian scale (Stages 0 to H). The lower limit of 7 years has been chosen based on the earliest reported onset of third molar formation, while the upper limit of 24 years corresponds to the age by which third molar root development is generally complete in most individuals, as supported by existing literature. (11) Eligible participants were required to have at least one visible developing third molar on an OPTG. OPTGs had to be of high diagnostic quality, with clear visibility of the crown and root morphology, allowing accurate staging according to the modified Demirjian method, and obtained from the same device (Planmeca Oy, Helsinki, Finland).

Exclusion criteria comprised history of orthodontic treatment, surgical procedures affecting the jaws, craniofacial trauma, presence of systemic or syndromic conditions known to influence dental development (e.g., endocrine disorders, metabolic syndromes, or genetic abnormalities), radiographic artifacts or anatomical overlap compromising visualization of the third molar region, incomplete dental records, pathological lesions in the area of interest, or radiographic evidence of third molars that could not be reliably staged. These exclusions were implemented to reduce confounding factors and ensure the internal validity and reproducibility of the developmental assessments.

-Dental Maturation Assessment

Third molar development was assessed using the modified Demirjian method, which classifies the dental maturation process into nine stages (Stage 0 to Stage H), based on the degree of crown and root formation. (7 , 12) All radiographic assessments were performed independently by an oral and maxillofacial radiologist, with over five years of experience.

-Data Collection and Variables

For each third molar, the Demirjian developmental stage was recorded along with the patient's chronological age, sex, jaw location (mandible or maxilla), and impaction-related anatomical classifications according to the Pell-Gregory and Winter systems.

-Statistical Analysis

Statistical analyses were conducted using JASP version 0.19.2 statistical software (University of Amsterdam, Amsterdam, The Netherlands). The distribution of the age and Demirjian staging variables were assessed for normality using descriptive statistics, histogram and Q-Q plots. Descriptive statistics were used to summarize demographic characteristics and the distribution of developmental stages. Normality of age distribution was assessed using the Shapiro-Wilk test. Skewness and kurtosis values were calculated to further evaluate the shape of the distribution.

The Kruskal-Wallis test was used to compare age across Demirjian stages, and Spearman's rank correlation coefficient was calculated to assess the association between chronological age and developmental stage. A generalized linear model (GLM) was further applied to evaluate the influence of age, sex, jaw, and impaction classifications on the developmental stage. A p-value of &lt;0.05 was considered statistically significant.

## Results

**Figure 1:**
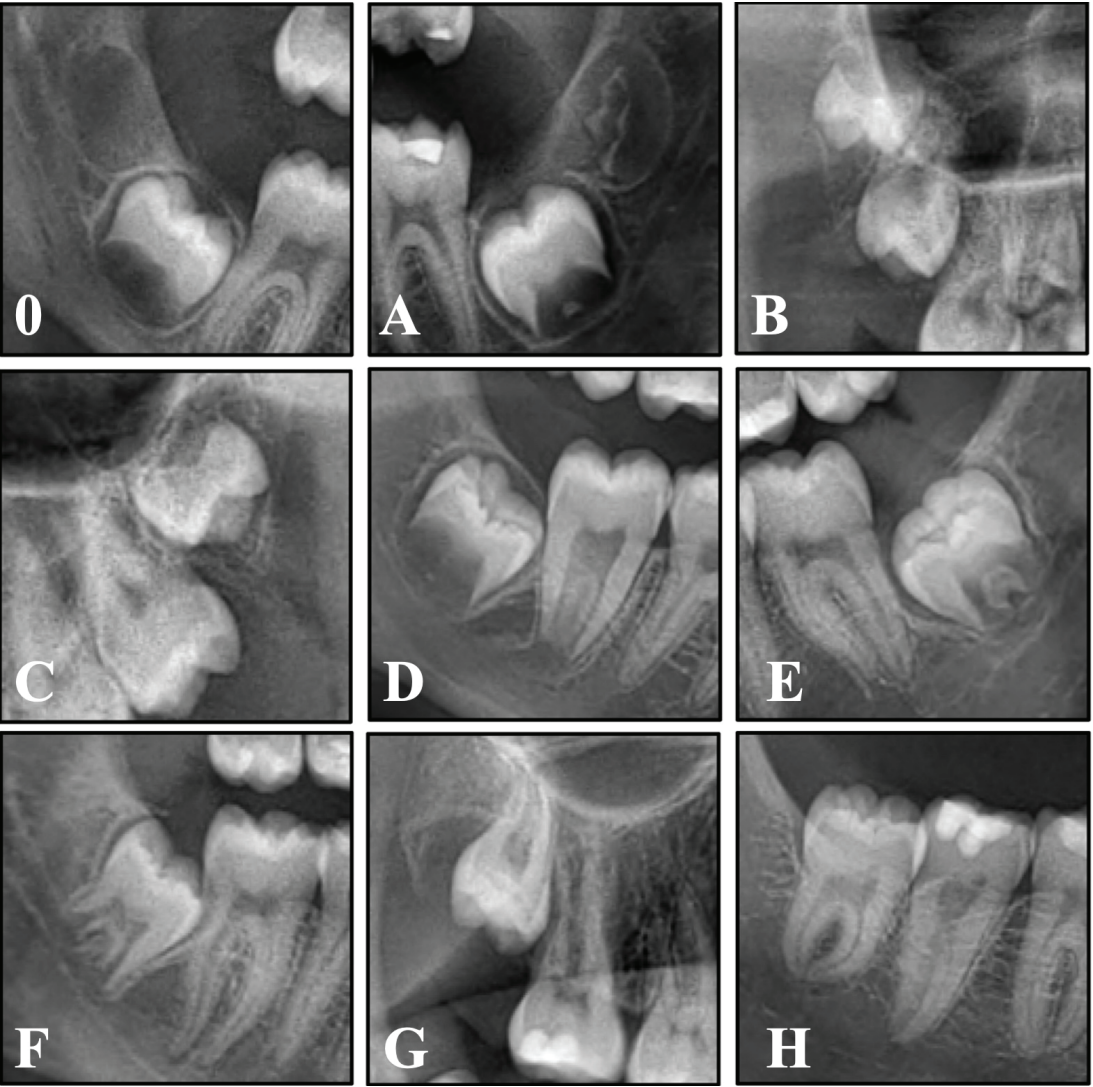
Examples of each Demirjian stage (0-H) from different quadrants.

A total of 1656 patients and 5323 third molar teeth (2770 mandibular, 2553 maxillary) were analyzed (Figure 1).

1

The mean chronological age of participants was 13.9 years (±3.78). Stage C (n=481) and stage E (n=470) were most frequently observed in mandibular third molars, while Stage C (n=507) and stage D (n=451) were more commonly encountered in maxillary third molars. Detailed contingency tables for mandibular and maxillary third molars according to age and modified Demirjian staging are shared in the supplementary document (Supplement 1 http://www.medicina.oral.com/carpeta/suppl1_27631).

The age and Demirjian staging distributions exhibited significant skewness indicating non-normality. In addition, the Shapiro-Wilk test yielded a statistically significant result (p&lt;0.001), further supporting the deviation from normality. Accordingly, non-parametric tests were employed for analyses involving age and Demirjian staging-related comparisons.

The Kruskal-Wallis test confirmed statistically significant differences in chronological age across all Demirjian stages for both mandibular and maxillary third molars (p&lt;0.001, Table 1) indicating a strong association between dental maturation and chronological age, but not with impaction-related anatomical classifications.

Table 1

Spearman correlation analysis demonstrated a strong positive association between chronological age and Demirjian stage for both mandibular and maxillary third molars (p&lt;0.001, Table 2).

Table 2

A generalized linear model was employed to evaluate the influence of age, gender, and impaction classifications (Winter and Pell and Gregory) on the Demirjian stage. The overall model demonstrated a statistically significant fit to the data (p&lt;.001), with pseudo-R² of 0.330 and 0.337 indicating that approximately 33% and 34% of the variability in the Demirjian stage may be explained by the included predictors for mandible and maxilla, respectively (Table 3).

Table 3

The omnibus tests from the generalized linear model revealed that age was a highly significant predictor of the Demirjian stage (p&lt;0.001). Similarly, the Winter classification demonstrated a statistically significant effect (p&lt;0.001). In contrast, gender (p=0.401) and Pell and Gregory classification (p=0.353) alone did not show significant main effects. Significant interaction effects were found for Pell-Gregory × Gender (p&lt;0.001) and Winter×Pell-Gregory (p=0.016), indicating potential moderating effects and context-specific developmental variation. Other interaction terms were not statistically significant (Table 4).

Table 4

## Discussion

This study provides robust evidence of a strong positive correlation between chronological age and third molar development, as assessed by the modified Demirjian method, in a large Turkish cohort. Our findings align with a growing body of literature supporting the validity of third molar developmental staging as a forensic age estimation tool, especially in populations undergoing prolonged dental maturation during adolescence and early adulthood (13 - 15).

The Spearman correlation coefficients observed (=0.858 for mandibular and =0.837 for maxillary third molars, p&lt;0.001) are among the highest reported for third molar-based age estimation in recent literature, underscoring the robustness of the modified Demirjian system in a Turkish context (7 , 14 , 16). The pronounced clustering of third molars within Stages B to E in our sample indicates a critical developmental window between the ages of 12 and 16 years, consistent with the known timing of third molar initiation and early root formation (11 , 17 , 18). This pattern reinforces the utility of third molars in estimating age within forensic and medico-legal thresholds, such as the differentiation between criminal responsibility thresholds at 12, 15, and 18 years in many jurisdictions (15 , 19).

The high statistical significance observed across Demirjian stages via the Kruskal-Wallis test (²=0.744 mandibular, ²=0.711 maxillary) confirms that molar development provides a well-differentiated scale for chronological stratification. However, the relatively modest R² values in the generalized linear models (0.330 and 0.337) highlight inherent biological variability, suggesting that the developmental stage of a third molar, though a significant predictor, is not sufficient as a standalone metric. This aligns with recent calls to integrate third molar development with additional biological indicators-such as cervical vertebral maturation or hand-wrist radiography-for multi-factorial age estimation protocols (20 , 21).

Notably, the interaction effects involving the Pell-Gregory and Winter classifications, particularly Pell-Gregory×Gender (p&lt;0.001) and Winter×Pell-Gregory (p=0.016), merit deeper consideration. These findings reflect complex anatomical and possibly hormonal influences on third molar maturation and impaction tendencies. While neither classification independently predicted developmental stage, their interaction with gender points to sex-specific skeletal and dental growth trajectories, potentially mediated by differences in mandibular growth rates, eruption space, and angulation dynamics (22). These findings are in line with recent cone-beam computed tomography (CBCT) based studies demonstrating differential mandibular volume expansion and eruption space by sex during adolescence (23 , 24).

From a clinical perspective, the anatomical classifications provide a useful proxy for surgical planning. However, their developmental implications-as evidenced by the interaction effects-suggest that these spatial metrics may also serve as early indicators of atypical molar development or eruption delay. This is particularly relevant for orthodontic decision-making regarding extraction timing or interceptive measures to prevent impaction-related pathologies (25).

Our data reinforce the notion that mandibular third molars tend to develop slightly earlier than maxillary ones, a pattern consistently reported in the literature and supported by histological evidence of earlier odontogenesis in the mandibular arch (26). This subtle temporal asymmetry can be exploited to refine age models, especially in borderline forensic cases.

The strengths of this study include its large, demographically diverse sample size, use of a well-validated developmental method, and simultaneous evaluation of anatomical and demographic variables. However, several limitations must be acknowledged. First, the cross-sectional design precludes assessment of individual developmental trajectories over time, limiting the ability to draw longitudinal inferences. Second, reliance on two-dimensional panoramic imaging, while widely accessible and cost-effective, may compromise staging precision compared to three-dimensional modalities such as CBCTs particularly in cases with overlapping anatomical structures (24 , 27). Future research should consider integrating multimodal imaging techniques with advanced computational methods, including machine learning algorithms, to improve the accuracy and objectivity of age estimation models. Furthermore, due to the potential influence of ethnic, genetic, and environmental factors on dental development, the generalizability of the present findings to other populations remains limited. The lack of external validation using independent or multi-center cohorts further constrains the applicability of these results beyond the studied Turkish sample. Longitudinal cohort studies incorporating diverse populations and imaging modalities are warranted to enable cross-population validation and enhance the robustness of forensic age estimation protocols.

## Conclusions

Third molar development, as assessed by the modified Demirjian method, remains a valuable and practical tool for age estimation in Turkish adolescents. However, its precision can be significantly enhanced through anatomical and demographic contextualization. The observed interaction effects emphasize the need for nuanced interpretation and pave the way for more sophisticated, individualized forensic models in the future.

## Figures and Tables

**Table 1:** Table Kruskal-Wallis analysis for age.

	χ²	df	p	ε²
DEMIRJIAN (Mandible)	2060	17	<.001	0.744
DEMIRIJAN (Maxilla)	1816	16	<.001	0.711

1

**Table 2:** Table Correlation analyses.

		AGE
DEMIRJIAN (Mandible)	Spearman's rho	0.858***
	DF	2768
	P-value	<.001
DEMIRJIAN (Maxilla)	Spearman's rho	0.837***
	DF	2551
	P-value	<.001

*p<.05, **p<.01, ***p<.001.

**Table 3:** Table Model fit.

	R²	df	X²	p
Mandible	0.330	27	3887	<.001
Maxilla	0.337	21	3497	<.001

3

**Table 4:** Table Overall omnibus tests.

	p
Age	<.001
Gender	0.401
Winter	<.001
Pell-Gregory	0.353
Gender ✻ Winter	0.905
Gender ✻Pell-Gregory	<.001
Winter ✻Pell-Gregory	0.016
Gender ✻ Winter ✻Pell-Gregory	0.080

4

## Data Availability

Declared none.

## References

[1] Gunst K, Mesotten K, Carbonez A, Willems G (2003). Third molar root development in relation to chronological age: A large sample sized retrospective study. Forensic Sci Int.

[2] Zandi M, Shokri A, Malekzadeh H, Amini P, Shafiey P (2015). Evaluation of third molar development and its relation to chronological age: A panoramic radiographic study. Oral Maxillofac Surg.

[3] Mincer HH, Harris EF, Berryman HE (1993). The A.B.F.O. study of third molar development and its use as an estimator of chronological age. J Forensic Sci.

[4] Galic I, Lauc T, Brkić H, Vodanovic M, Galić E, Biazevic M, Brakus I, Badrov J, Cameriere R (2015). Cameriere’s third molar maturity index in assessing age of majority. Forensic Sci Int.

[5] Schmeling A, Reisinger W, Geserick G, Olze A (2006). Age estimation of unaccompanied minors. Part I. General considerations. Forensic Sci Int.

[6] Khojastepour L, Khaghaninejad MS, Hasanshahi R, Forghani M, Ahrari F (2019). Does the Winter or Pell and Gregory classification system indicate the apical position of impacted mandibular third molars?. J Oral Maxillofac Surg.

[7] Demirjian A, Goldstein H, Tanner JM (1973). A New System of Dental Age Assessment. Human Biol.

[8] Galić I, Vodanović M, Cameriere R, Nakaš E, Galić E, Selimović E, Brkić H (2011). Accuracy of Cameriere, Haavikko, and Willems radiographic methods on age estimation on Bosnian-Herzegovian children age groups 6-13. Int J Legal Med.

[9] Nykänen R, Espeland L, Kvaal SI, Krogstad O (1998). Validity of the Demirjian method for dental age estimation when applied to Norwegian children. Acta Odontol Scand.

[10] (2014). Radiographic evaluation of third molar development in 6- to 24-year-olds. Imaging Sci Dent.

[11] Orhan K, Ozer L, Orhan AI, Dogan S, Paksoy CS (2007). Radiographic evaluation of third molar development in relation to chronological age among Turkish children and youth. Forensic Sci Int.

[12] Bijjaragi SC, Sangle VA, Saraswathi F, Patil VS, Ashwini Rani S, Bapure SK (2015). Age estimation by modified Demirjian’s method (2004) and its applicability in Tibetan young adults: A digital panoramic study. J Oral Maxillofac Pathol.

[13] Darıcı A, Ölmez MS, Güngör HC, Rajavaara P, Sipola A, Anttonen V, Päkkilä J (2024). Comparison of accuracy of different dental age estimation methods in Finnish and Turkish populations. Acta Odontol Scand.

[14] Sisman Y, Uysal T, Yagmur F, Ramoglu S (2007). Third-molar development in relation to chronologic age in Turkish children and young adults. Angle Orthod.

[15] Duruk G, Gündogdu Özdal T, Duman S (2022). Accuracy of age estimation with Demirjian and Nolla methods in Eastern Turkish children aged 3-17 years old. Eur Oral Res.

[16] Milani S, Shahrabi M, B Fakhar H, Parvar S, Abdolahzadeh M (2022). Accuracy of Demirjian’s and Cameriere’s Methods for Age Estimation in 6- to 10-Year-Old Iranian Children Using Panoramic Radiographs. Int J Dent.

[17] Pillai JP, Nilendu D, Thomas N, Nagpal S, Sneha Nedunari LS (2021). Inter-observer agreement in the radiographic interpretation of Demirjian’s developmental stages in the mandibular second and third molars - A comparative study. J Oral Maxillofac Pathol.

[18] Timme M, Bender J, Steffens L, Shay D, Schmeling A (2023). Third Molar Eruption in Dental Panoramic Radiographs as a Feature for Forensic Age Assessment-Presentation of a New Non-Staging Method Based on Measurements. Biology.

[19] Kaka RAE, Haiba MIM, Sheta AAE, Salama NH, Enany NM (2025). Age estimation using dental and hand-wrist radiography among a sample of Egyptian children. Sci Rep.

[20] Márquez-Ruiz AB, González-Herrera L, Luna J de D, Valenzuela A (2025). Radiologic assessment of third molar development and cervical vertebral maturation to validate age of majority in a Mexican population. Int J Legal Med.

[21] Guo X, Gao Y, Zhang F, Wang M, Tian X, Huang Q, Liu X (2023). Assessment of mandibular retromolar space in adults with regard to third molar eruption status. Clin Oral Investig.

[22] Mubarak H, Tajrin A, Gazali M, Rahman FUA (2024). Impacted mandibular third molars: A comparison of orthopantomography and cone-beam computed tomography imaging in predicting surgical difficulty. Arch Craniofac Surg.

[23] Behbehani F, Årtun J, Thalib L (2006). Prediction of mandibular third-molar impaction in adolescent orthodontic patients. Am J Orthod Dentofacial Orthop.

[24] Huang Y, Chen Y, Yang D (2023). Three-dimensional analysis of the relationship between mandibular retromolar space and positional traits of third molars in non-hyperdivergent adults. BMC Oral Health.

[25] Gamba TDO, Alves MC, Haiter-Neto F (2016). Mandibular sexual dimorphism analysis in CBCT scans. J Forensic Leg Med.

[26] Ajmal MA, Roberts TS, Beshtawi KR, Raj AC, Sandeepa NC (2023). Sexual dimorphism in odontometric parameters using cone beam CT: A systematic review. Head Face Med.

[27] Carter K, Worthington S (2016). Predictors of Third Molar Impaction: A Systematic Review and Meta-analysis. J Dent Res.

